# Assessing the Social Determinants of Health and Adverse Childhood Experiences in Patients Attending a Children's Hospital Cleft Palate-Craniofacial Program

**DOI:** 10.1177/10556656211048742

**Published:** 2021-11-03

**Authors:** Ethan Ponton, Rebecca Courtemanche, Tanjot K. Singh, Damian Duffy, Douglas J. Courtemanche, Christine Loock

**Affiliations:** 1 Office of Pediatric Surgical Evaluation and Innovation, 12358 British Columbia Children’s Hospital, University of British Columbia, Vancouver, British Columbia, Canada; 2 Department of Orthopaedics, 8166 University of British Columbia, Vancouver, British Columbia, Canada; 3 Department of Surgery, 8166 University of British Columbia, Vancouver, British Columbia, Canada; 4 Division of Plastic Surgery, British Columbia Children’s Hospital, Vancouver, British Columbia, Canada; 5 Department of Pediatrics, 8166 University of British Columbia, Vancouver, British Columbia, Canada; 6 Department of Pediatric Surgery, 8166 University of British Columbia, Vancouver, British Columbia, Canada; 7 Department of Pediatrics, 37210 British Columbia Children’s Hospital, Vancouver, British Columbia, Canada

**Keywords:** quality of life, Social support, Pediatrics

## Abstract

**Objective:**

This study aimed to describe the social determinants of health (SDoH) for patients receiving multidisciplinary team care in a Cleft Palate-Craniofacial program, develop responsive and consistent processes to include trauma-informed psychosocial histories, promote discussions about additional “non-medical” factors influencing health and surgical outcomes, and demonstrate that these activities are feasible in the context of multidisciplinary patient-provider interactions.

**Design:**

Single-site, cross-sectional study using a questionnaire.

**Setting:**

Participants were recruited from a provincial quaternary care Cleft Palate-Craniofacial program at British Columbia Children's Hospital in Vancouver, BC, Canada.

**Participants:**

290 families completed the questionnaire.

**Results:**

34% of families experience significant barriers to accessing primary health care, 51% struggle financially, and 11% scored four or more on the Adverse Childhood Experiences scale. Furthermore, 47% reported not having adequate social support in their lives, and 5% reported not feeling resilient at the time of the survey.

**Conclusion:**

Patients with cleft and craniofacial anomalies have complex needs that extend beyond the surgical and medical care they receive. It is critical that all Cleft and Craniofacial teams incorporate social histories into their clinic workflow and be responsive to these additional needs. Discussions surrounding SDoH and adversity are welcomed by families; being involved in the care and decision-making plans is highly valued. Healthcare providers can and should ask about SDoH and advocate for universal access to responsive, site-based, social work support for their patients.

## Introduction

The World Health Organization (2021) defines social determinants of health [SDoH] as the non-medical factors that influence health, and it is well known that people in a lower socioeconomic position have poorer health outcomes. The King’s Fund (2021) and Braveman and Gottlieb (2014) estimate SDoH have a greater impact on health than medical care, especially when determining who gets sick in the first place. Furthermore, social factors are comparable to pathophysiological causes of death in the United States (Galea et al., 2011). With respect to surgical treatment, SDoH should be considered pre- and post-operatively, as those who lack resources are less likely to be able to follow treatment plans and are at higher risk of readmission (Kangovi and Grande, 2011; Lax et al., 2017). This places an unnecessary burden on both the patient and the healthcare system.

Canada has one of the highest orofacial cleft rates in the world at approximately 1 in 790 live births (Pavri and Forrest, 2013). There are 21 cleft centers in Canada. Our program has over 2400 active patients in a province with approximately 1 million children and youth (Canadian Child Welfare Research Portal, 2019). Patients require care from a wide variety of specialists, including nursing, pediatrics, genetics, otolaryngology, plastic surgery, social work, psychology, audiology, speech-language pathology, orthodontics and dentistry (Robin et al., 2006; Seattle Children’s Hospital, 2018). Current literature describes the social and psychological implications of undergoing reconstructive surgery for cleft and/or craniofacial abnormalities in children (Sousa et al., 2009; Bemmels et al., 2013; Glener et al., 2017; Al-Namankany and Alhubaishi, 2018); however, there are also psychosocial and socioeconomic implications of cleft care for both the child and caregivers. For example, the child may struggle with discontent with their appearance, teasing or peer acceptance, abuse/neglect, and learning and behaviour problems, whereas the parent may struggle with securing community and financial resources (Seattle Children’s Hospital, 2018).

Although the psychosocial and socioeconomic needs of patients with orofacial clefts have been previously described, there is limited information on SDoH for this patient population. Therefore, we sought to describe the SDoH for patients attending the British Columbia Children's Hospital (BCCH) Cleft Palate-Craniofacial program and determine if it is possible for the Cleft Palate-Craniofacial healthcare team to discuss SDoH with patients and families in a trusted and responsive environment.

## Methods

### Study Design

A quality improvement study was conducted with all patients attending the BCCH Cleft Palate-Craniofacial program over a 2-year period, between January 2018 and February 2020 (pre-COVID-19 restrictions). Quality improvement study approaches were recommended by the University of British Columbia Research Ethics Board due to perceived sensitivity and possible need for immediate child protection reporting and intervention (e.g. responses regarding duty to report concurrent child maltreatment potentially arising from an ACEs Questionnaire). Quality improvement activities are exempt from Research Ethics Board review as stated in the ethics framework of article 2.5 of the TCPS2 for research involving human participants. The project protocol was reviewed and approved by our institution's Research Privacy Officer.

### Survey Development

The first survey used in this study, the Surgery and Society Questionnaire (SASQ) (supplemental file 1) included questions related to demographics, social and material capital, healthcare utilization and access, food security, housing security, financial security, and adversity and resiliency factors. Survey development was also guided by concurrent collaboration with and use of the BC Divisions of Family Practice and their Poverty Intervention tool (Centre for Effective Practice, 2016) and Brcic et al.'s poverty screening study (Brcic et al., 2011). The survey included the validated Adverse Childhood Experiences Questionnaire (Burke Harris and Renschler, 2015) as well as an associated Resiliency Questionnaire (Rains et al., 2006). Consultations with clinicians from the departments of Social Pediatrics, Social Work, and Surgery led to the development of additional novel questions based on earlier research and clinician expertise (Wong et al., 2012). More direct questions on race, ethnicity, and Indigenous identity were included in the SASQ based on the 2016 Census of Population questions, long form (Statistics Canada, 2016)**.** These were abbreviated and revised based on input from our institutional indigenous scholar and ethicist, shortening and adding calls to actions from the Truth and Reconciliation Commission of Canada (2015) #19 and #20 regarding “closing gaps” [heath disparities] and addressing barriers to care and “the distinct health needs of the Métis, Inuit, and off-reserve Aboriginal peoples.” Other questions were developed to obtain additional socioeconomic data, characterized by income, occupation, and education (Bradley and Corwyn, 2002). Open ended questions were included based on initiating and improving “What Matters to You BC” conversations based on patient and family centred priorities (WMTYBC, 2021).

After one year, preliminary data were reviewed to shorten the questionnaire for use in a busy interdisciplinary clinical setting. The questions most likely to indicate a family was in need were identified and formed our BEARS questionnaire (supplemental file 2), which included five key categories: **B**arriers to care, **E**conomic factors, **A**dversity, **R**esiliency, and **S**ocial capital.

### Data Collection and Analysis

All families who came to the BCCH Cleft Palate-Craniofacial clinic were given the option to complete a paper copy of the questionnaire at check-in and were given an opportunity to ask questions. Exclusion criteria included families who relied on interpreter services and children in government care without their legal guardian present. The surveys were completed by the parent/guardian, and the completed questionnaires were reviewed by a clinician and/or social worker with each family during their appointment. Concerning responses were highlighted as “Red Flags” and received appropriate clinical follow-up with the team pediatrician, nurse clinician/care coordinator, and/or social worker. Responses were entered into the Research Electronic Data Capture (REDCap) tool hosted at the BC Children's Hospital Research Institute (Harris et al., 2009, 2019), and tabulated using Microsoft Excel. Continuous variables were summarized by medians and interquartile ranges and categorical variables were summarized by frequencies and percentages. Where applicable, responses were grouped and compared using independent samples t-tests, chi-squared tests, or by one-way ANOVA. Significant ANOVAs were followed by post-hoc testing using Tukey’s HSD test. A significance level of 0.05 was used for all statistical tests. All data were analyzed using Microsoft Excel or JASP (Version 0.14.1) (JASP Team, 2020).

## Results

### Demographics

In total, 290 families out of 604 families completed the surveys (48% response rate): 131 families completed the SASQ, and 159 families completed the BEARS questionnaire. The respondents for each questionnaire version did not differ in terms of number of people living in the home, number of children living in the home, nor identity as a minority (Table 1).

**Table 1.** Demographics.

	Surgery and Society (N = 131)	BEARS (N = 159)	p-value
How many people live in your home?^1^	4 [4, 5]	4 [4, 5]	0.68^2^
How many children live in your home?^1^	2 [2, 3]	2 [2, 3]	0.38^2^
Do you identify as First Nations, Metis, Inuit, or Indigenous?	26 (20%)	N/A	N/A
Does anyone in your household identify as a minority?			
Yes	39	37	0.63^3^

^1^Median [IQR]; ^2^Independent Samples t-test; ^3^χ^2^ Contingency test.

### Barriers to Care

Overall, 34% (96/284) of participants reported that they either do not have a regular healthcare provider, or if they do, they cannot turn to them for additional assistance for help with disability forms, nutrition supplements, housing and transportation-related forms, or other related issues; 21% (27/131) reported having difficulties seeing a healthcare provider for health-related concerns. In patient-provider interactions, 59% (77/131) of families reported that it is important their provider ask about financial and social stressors in their lives, and almost all (97%, 274/282) reported that it is important for their provider team to include them in the decision-making plans about their child's health (Table 2).

**Table 2.** Barriers to Care.

Questions	Response Options	Count/Total (%)
If you have a family doctor, nurse practitioner, counselor, or other general health provider, can you turn to them for support for help with disability forms, nutrition/caloric supplements (eg Vitamins/Boost), housing, transportation-related forms, etc.?	Yes	188/284 (66%)
	No	73/284 (26%)
	I don't have a regular health provider	23/284 (8%)
How important is it to you to have your health provider team (nurse, doctor, nurse practitioner) ask about financial or other social stresses in your life?	Very important	35/131 (27%)
	Somewhat important	42/131 (32%)
	Neutral	37/131 (28%)
	Not Important	13/131 (10%)
	Not ever an issue	4/131 (3%)
How important is it that your health care provider include you in the decisions about your child's health?	Very important	257/282 (92%)
	Somewhat important	17/282 (6%)
	Neutral	4/282 (1%)
	Not Important	0/282 (0%)
	Not ever an issue	4/282 (1%)
How easy is it for you to see a doctor or other healthcare provider for health care concerns?	Very difficult	4/131 (3%)
	Difficult	23/131 (18%)
	Neutral	42/131 (32%)
	Easy	53/131 (40%)
	Very easy	9/131 (7%)

### Economic Factors

28% (75/269) of participants reported an estimated household income of less than $40,000 CDN, and the number of people living in the home did not differ by income, *F*(3, 262) = 0.327, p = 0.81. Of participants who identified as First Nations, Metis, Inuit, or Indigenous, 65% (15/23) reported an estimated household income of less than $40,000 compared to 17% (18/108) in the rest of the sample. In addition, from data collected from the 131 patients who completed the SASQ, 26% (34/130) of parent respondents reported having a high school diploma or less, 26% (34/130) a non-university certificate or diploma, 34% (44/130) a university certificate or diploma, and 14% (18/130) an advanced degree. While 99% (130/131) of families reported that they have a secure and stable place to live, 25% (32/130) indicated they do not always have access to nutritious foods for their family, and 51% (145/284) always or sometimes have trouble making ends meet at the end of the month. Using the merged data sets (SASQ and BEARS), 14% (40/278) reported finding the cost of supplies and treatment plans prohibitive. Overall, 49% (135/277) of families routinely submit their tax forms to be considered for benefits, and families with lower incomes were more likely to submit forms for benefits compared to those with higher income brackets, *χ^2^*(6, N = 266) = 26.2, p < 0.001 (Table 3).

**Table 3.** Economic Factors.

Questions	Response Options	Count/Total (%)
What is your estimated annual household income?	$0-$40,000	75/269 (28%)
	$40,000-$80,000	73/269 (27%)
	$80,000-$120,000	69/269 (26%)
	$120,000 and over	52/269 (19%)
What is your highest level of formal education?	Advanced Degree (ie Master's, JD, MD, PhD)	18/130 (14%)
	University certificate or diploma: Bachelor's	26/130 (20%)
	University certificate or diploma below bachelor level	18/130 (14%)
	Apprenticeship or other trades certificate or diploma	6/130 (4%)
	CEGEP or other non-university diploma	28/130 (22%)
	Secondary/high school diploma	26/130 (20%)
	Some high school	7/130 (5%)
	Less than high school	1/130 (1%)
Do you have access to a secure and stable place to live?	Yes	130/131 (99%)
	No	1/131 (1%)
Do you feel you have access to nutritious foods for your family?	Always	98/130 (75%)
	Sometimes	31/130 (24%)
	Never	1/130 (1%)
At the end of the month, do you have difficulty making ends meet?	Always	27/284 (9%)
	Sometimes	118/284 (42%)
	Never	139/284 (49%)
Does the cost of essential medication, devices, disposables, or other medical supplies affect your ability to follow prescribed treatment plans, or provide for your child?	Yes	40/278 (14%)
	No	238/278 (86%)
Do you submit tax forms each year to be considered for your child or disability eligibility?	Yes	135/277 (49%)
	No	101/277 (36%)
	Unsure	41/277 (15%)

### Adversity

Regarding the optional section on ACEs, 75% (217/290) of families responded.

11% (23/217) of participants had an ACE score of four or more (Table 4). There was a statistically significant effect of household income on ACE score, *F*(3, 205) = 5.94, p < 0.001, ω^2^ = 0.066. Post-hoc comparisons using Tukey's HSD test revealed statistically significant mean differences between the lowest income group, $0-$40,000, and both the $80,000-$120,000 and $120,000 + groups, where the lowest income group had a significantly higher mean ACE score than the higher income groups.

**Table 4.** Adversity.

Questions	Response Options	Count/Total (%)
Count the number of statements that apply to your child and indicate the total number: At any point since your child was born…	0	95/217 (44%)
	1	57/217 (26%)
	2	29/217 (13%)
	3	13/217 (6%)
	4	14/217 (6%)
	5	6/217 (3%)
	6	0/217 (0%)
	7	1/217 (1%)
	8	2/217 (1%)
	9	0/217 (0%)
	10	0/217 (0%)

### Resiliency and Social Capital

47% (124/264) of respondents reported that they rarely or only sometimes have adequate social support. In addition, 34% (96/286) of families reported having fewer than four people to turn to for support in times of stress and 5% (15/290) of participants indicated that they weren't feeling resilient at the time of their appointment (Table 5).

**Table 5.** Resiliency and Social Capital.

Questions	Response Options	Count/Total (%)
Do you feel there is a supportive network available to help you or your family members in times of need, if needed, at this hospital or nearby? (eg spiritual care, quiet spaces, healing circle, social work support, patient navigators, interpreters, etc)	Often	140/264 (53%)
	Sometimes	100/264 (38%)
	Rarely	24/264 (9%)
In times of stress, how many people can you turn to for support? (eg friends, partner, parents, grown children, neighbours, elders, spiritual/religious guide, teacher, coach, health nurse, doctor, co-worker, etc)	Fewer than 4	96/284 (34%)
	4–8	114/284 (40%)
	9–13	25/284 (12%)
	14–19	14/284 (5%)
	More than 20	25/284 (9%)
I am having a hard time feeling resilient right now	Checked	15/290 (5%)

## Discussion

The results of this study show that the needs of the Cleft Palate-Craniofacial population extend beyond the medical care that they receive within the walls of the hospital. By offering an optional survey to each family at the beginning of clinic, we were able to initiate discussions that enabled us to better understand the SDoH in each family and in our patient population. Though these conversations might have been foreseen as uncomfortable, the majority of families responded that it is important for their healthcare team to ask about financial and social stressors in their lives, and they welcomed these conversations as evidenced both in their open comments and rates of questionnaire completion. They also valued being engaged in discussions about care plans for their children. However, significant barriers to accessing care outside our clinic remain, with one third of families lacking an engaged primary healthcare provider or “medical home” (Hefford, 2017). Many families experience both material and social poverty, including food and financial insecurity, with half reporting lack of consistent social support, half having trouble making ends meet, and one quarter lacking consistent access to nutritious foods for their family. Furthermore, we found that income may buffer adversity: those in the lowest household income group had significantly higher ACE scores than the two highest income groups. Importantly, though participants were reminded that responding to the ACEs question was optional, three quarters of participants responded, indicating these questions are generally accepted and not too sensitive to ask, and families are comfortable and willing to share this information with their care team in the right setting. As one young adult responded to a pediatrician author on our team when asked about answering questions on the ACEs, “Of course you should know these things. You are not some random. You are my doctor.”

Compared to both the Canadian and provincial average rates of child poverty (18.2% and 18.5% respectively), our clinic poverty rate is considerably higher (FCBCCYAC, 2020). Indigenous identifying families also accounted for a disproportionate percentage reporting living on an estimated household income of under $40,000. Indigenous families (including First Nations, Metis, Inuit, and others identifying as Indigenous) made up 20% of our study population and are over-represented in our clinical population. This has been well described in prior population-based health surveillance registry studies ascertaining a higher incidence of orofacial clefts (3.74 per 1000 live births for Indigenous vs total rate of 1.97 per 1000 live births for the combined provincial population (Lowry and Renwick, 1969; Lowry and Trimble, 1977; Lowry et al., 2009).

Our results also suggest that higher incomes are associated with decreased adversity and a lower number of cumulative ACEs. Halfon et al. (2017) highlighted that while income is not necessarily protective against adversity, there was a “steep income gradient, particularly for children who experienced ≥4 ACEs.” In agreement with our results, they found higher income groups were less likely to report ACEs than those of lower income groups. While ACEs are less common in higher income groups, ACEs overall may have a greater influence on health outcomes (Halfon et al., 2017). Therefore, while income may decrease the occurrence or reporting of ACEs, it may not protect against the negative health effects of childhood adversity. Furthermore, higher ACEs are associated with an increased likelihood of not completing high school, unemployment, and decreased income potential (Metzler et al., 2017), initiating or propagating a cycle, making intergenerational mobility increasingly difficult.

Resiliency and social support are critical but often overlooked determinants of health. Low social support is related to higher medical morbidity and mortality, whereas high social support may be protective for mental and physical health (Southwick et al., 2004). In addition, the seminal Werner (1993) Kauai Longitudinal Study found that children who were exposed to biological and psychosocial risk factors and exhibited resiliency were more successful in their transition to adulthood than their low-resiliency, high-risk peers. Intrinsic resiliency as well as protective factors (caregiving styles that fostered self-esteem, supportive adults, developing skills that allowed them to use the abilities they had) allowed them to achieve educational and vocational accomplishments equal to those of the low-risk cohort (Werner, 1993).

### Limitations

Although the survey was offered to every family in clinic with the few exceptions noted, self-selection bias was likely present as the surveys were optional. Additionally, we did not track how many families declined to answer the questionnaires. We relied on self-reporting, so recall bias was an inherent limitation. In addition, when transitioning from the SASQ to the BEARS questionnaire, twelve questions were excluded or re-worded. For the questions that had a slight re-wording, the observed distributions were very similar, providing confidence that they could be analyzed together. When transferring to the BEARS questionnaire, one of the options for the ACEs question—"Your child lived with someone who had a problem with drinking or using drugs”—was missed, so the frequencies for ACEs may be underrepresented. The surveys used in this study have not been validated; however, survey validation was not our objective, as we wanted to use the questionnaires to take a consistent and trauma-informed social history addressing SDoH and gaps in health care access, and if feasible, ask safely about ACEs. We expect other clinics who use our questionnaire to adapt questions to meet the needs of their own clinical setting.

### Future Directions

Many families in our clinic continue to experience material and social poverty and low resiliency. Though recruitment for this phase of our QI study was concluding as the COVID-19 pandemic started, we were able to pivot to develop an on-line REDCap Questionnaire for families and have added a 5-point BEARS-like “COVID Check-in,” based on practice point recommendations from the Canadian Paediatric Society Social Paediatrics Section (Suleman et al., 2020). More recently, Li et al. (2021) found that while both social support and resiliency are protective factors for mental health during the pandemic, perceived social support has declined due to forced isolation. Therefore, asking patients and families about social support and resiliency are even more important during situations in which individuals or families may be more socially isolated.

The survey needs to be further refined to a manageable number of questions that accurately describe a patient's SDoH without overburdening the patients, families, and staff in a busy surgical clinic setting. Care teams that want to use the questionnaire need to be educated about “red flag” answers so that critical issues do not go unnoticed or unattended. It is imperative that there is a hospital-supported social safety net of social workers that can support patients, liaise with community supports, and provide immediate help and assistance where needed (eg, travel and food vouchers). Ongoing social awareness needs to be raised within the medical staff and hospital community about the issues for patients related to their SDoH to make this part of routine assessment and care. Ultimately, the goal is for these questionnaires to enable the practice of taking a more actionable social history and to ask and to respond to historical and concurrent ACEs.

## Conclusion

In conclusion, the Cleft Palate-Craniofacial patient population is complex, with diverse profiles of SDoH. It is well known that SDoH may impact quality of life as well as health outcomes, and these factors should not be ignored when providing medical care. With our survey, we are able to describe two years of data involving the views of 290 families receiving direct care from our program. Our study has demonstrated that discussions surrounding SDoH and adversity are welcomed by families who value being involved in their child's care and decision-making plans. It is critical that all Cleft and Craniofacial teams incorporate more standardized, culturally safe, trauma-informed, child- and family-centered social histories into their clinic workflow and be responsive to patients’ additional needs. Healthcare providers can and should ask about SDoH and advocate for social work support for their patients. Through respectful discussions and understanding, the BEARS questions may improve patient-provider interactions, clinical care, and ultimately, the health outcomes of patients.

Epilogue

“Universal access to high quality care and a focus on equitable outcomes, then, is central to challenging health inequities. So too is challenging inequities in social conditions which lead to health inequalities” (Marmot and Allen, 2014).

Studies such as this highlight the need for programs to collect similar SDOH data. After 2 decades of documenting and explicating the evidence for SDOH relating to increasing health disparities for our Cleft Palate-Craniofacial population, essential additional social work, speech, and nursing resources for our provincial Cleft Palate-Craniofacial interdisciplinary program have recently been prioritised through our publicly funded provincial health authority to ensure and improve timely and universal access to CPCF team care.

## Supplemental Material

sj-docx-1-cpc-10.1177_10556656211048742 - Supplemental material for Assessing the Social Determinants of Health and Adverse Childhood Experiences in Patients Attending a Children's Hospital Cleft Palate-Craniofacial Program Click here for additional data file. Supplemental material, sj-docx-1-cpc-10.1177_10556656211048742 for Assessing the Social Determinants of Health and Adverse Childhood Experiences in Patients Attending a Children's Hospital Cleft Palate-Craniofacial Program by Ethan Ponton, Rebecca Courtemanche, Tanjot K. Singh, Damian Duffy, Douglas J. Courtemanche and Christine Loock in The Cleft Palate-Craniofacial Journal

sj-docx-2-cpc-10.1177_10556656211048742 - Supplemental material for Assessing the Social Determinants of Health and Adverse Childhood Experiences in Patients Attending a Children's Hospital Cleft Palate-Craniofacial Program Click here for additional data file. Supplemental material, sj-docx-2-cpc-10.1177_10556656211048742 for Assessing the Social Determinants of Health and Adverse Childhood Experiences in Patients Attending a Children's Hospital Cleft Palate-Craniofacial Program by Ethan Ponton, Rebecca Courtemanche, Tanjot K. Singh, Damian Duffy, Douglas J. Courtemanche and Christine Loock in The Cleft Palate-Craniofacial Journal
