# Supplementary material for: Assessing the Social Determinants of Health and Adverse Childhood Experiences in Patients Attending a Children's Hospital Cleft Palate-Craniofacial Program
Source: Cleft Palate Craniofac J. 2021 Nov 3;59(12):1482–9. doi: 10.1177/10556656211048742 (PMC9585543; doi:10.1177/10556656211048742)
Supplement: sj-docx-1-cpc-10.1177_10556656211048742 - Supplemental material for Assessing the Social Determinants of Health and Adverse Childhood Experiences in Patients Attending a Children's Hospital Cleft Palate-Craniofacial Program [file sj-docx-1-cpc-10.1177_10556656211048742.docx]

**Surgery and Society Questionnaire**

Do you have a family doctor, nurse practitioner, counselor, or other health provider?

- Yes
- No

Can you turn to them for assistance (such as disability, nutritious supplements, housing, transportation, etc.)?

- Yes
- No

Do you ever ask anyone to come along with you to your child’s appointment and help?

- Yes
- No

Do you believe you have an adequate social network for encouragement or support?

- Never
- Sometimes
- Always

How important is it to you to have your health provider team (nurse, doctor, nurse practitioner) ask about financial or other social stresses in your life?

- Very important
- Somewhat important
- Neutral
- Not important
- Not ever an issue

How easy is it for you to see a doctor or other healthcare provider for health care concerns?

- Very difficult
- Difficult
- Neutral
- Easy
- Very east

How important is it for them to include you in the decision making treatment plans?

- Very important
- Somewhat important
- Neutral
- Not important
- Not ever an issue

How important is it to you to have members of your health provider team (nurse, doctor) treat you like an equal?

- Very important
- Somewhat important
- Neutral
- Not important
- Not ever an issue

How important is it for the medical office assistant staff to get to know about you and your family situation?

- Very important
- Somewhat important
- Neutral
- Not important
- Not ever an issue

*The purpose of these questions is to understand your family’s basic information. It does not include any specific identifying information. The reason we ask about postal code, is that children at our hospital come from all over the providence, and we want to know how representative our project sample is of the population of British Columbia.*

How many people live in your home? __________

How many children (<19yo) live in your household? __________

What are the first 3 characters of your postal code? __________

*We are interested in knowing if families across the province have difficulties in their ability to access foods and housing. If there is a difference, we hope to use this data to advocate for better access to healthy foods and safe housing.*

Do you feel you have access to nutritious foods for your family?

- Never
- Sometimes
- Always

Do you have access to a secure and stable place to live?

- Yes
- No
- Sometimes

*It is recognized that 1 in 5 children in British Columbia is experiencing some form of poverty. Financial strain can impact the care of children, including problems being able to access healthcare or to purchase medications. The purpose of these questions is to understand how many children admitted to this hospital may be experiencing financial poverty.*

Do you have difficulties making ends meet at the end of the month?

- Never
- Sometimes
- Always

Have you completed and sent in your tax forms to be considered for benefits?

- Yes
- No
- Unsure

Does the cost of essential medicines, devices, disposables, or other medical supplies ever affect your ability to follow treatment plans?

- Yes
- No

How does the cost of essential medicines, devices, disposables, or other medical supplies affect you or your ability to follow treatment plans? _________________________________________

Do you receive any of the following?

- Federal Child Tax Benefit
- Federal Disability Child Tax Benefits (T2201 Disability Tax Credit Certificate filled out by a doctor or other special needs provider)
- Persons with disabilities (PWD) assistance for the province
- First Nations Health Benefits
- Other payments for a disability (such as ICBC payments, worker’s compensation, etc.)
- Extra-income supplements
- Other __________

Do you identify as any of the following? Please check one or more.

- First Nations
- Metis
- Inuit
- Indigenous
- Other __________
- None of these

*Please note that the following questions are more sensitive. If at any time you feel uncomfortable and would like to stop, or withdraw your consent from the project as a whole, please indicate this to the Research Assistant.*

*There are many experiences that can increase stress in children, potentially leading to adverse health outcomes. A group of experts has determined a set of experiences that they think are likely to contribute to childhood social adversity. The purpose of this section is to determine how many children admitted to this unit have had any of these experiences. “ACEs” are Adverse Childhood Experiences that may negatively affect the health of an individual.*

Of the following statements, how many of these “ACEs” apply to your child? __________

At any point since your child was born…

- Your child’s parents or guardians were separated or divorced
- Your child lived with a household member who served time in jail or prison
- Your child lived with a household member who was depressed, mentally ill, or attempted suicide
- Your child saw or heard household members hurt or threated to hurt each other
- A household member swore at, insulted, humiliated, or put down your child in a way that scared your child OR a household member acted in a way that made your child afraid that s/he might be physically hurt
- Someone touched your child’s private parts or asked to touch their private parts in a sexual way
- More than once, your child went without food, clothing, a place to live, or had no one to protect her/him
- Someone pushed, grabbed, slapped, or threw something at your child OR your child was hit so hard that your child was injured or had marks
- Your child lived with someone who had a problem with drinking or using drugs
- Your child often felt unsupported, unloved, and/or unprotected

Do you or anyone in your household identify as a minority visually or culturally?

- Yes
- No

*Socioeconomic status can give researchers and health care providers important insight into a family’s life. SES can, in general, be estimated as a combination of education, occupation, and income. The following questions will allow the research team to approximate socioeconomic status.*

What is your highest level of formal education?

- Less than high school
- Some high school
- Secondary/high school diploma
- Apprenticeship or other trades certificate or diploma
- CEGEP or other non-university certificate or diploma
- University certificate or diploma below bachelor level
- University certificate or diploma: Bachelor’s
- Advanced degree (i.e. Masters, JD, MD, PhD)

Do you work outside the home?

- Yes
- No

What is your estimated annual household income?

- $0-20,000
- $20,000-$40,000
- $40,000-$60,000
- $60,000-$80,000
- $80,000-$100,000
- $100,000-$120,000
- $120,000 +

*Having supports in the family and community can help families overcome adversity and develop resiliency. The following questions ask about the supports in your life.*

In times of stress, how many people can you turn to for support (i.e. friends, partner, parents, gown children, neighbours, elder, spiritual/religious guide, teacher, coach, health nurse, doctor, co-worker, etc.)?

- < 4
- 4-8
- 9-13
- 14-19
- > 20

Which of the following apply to you?

- When I was little, other people helped my parents take care of me and they seem to love me
- I've heard that when I was an infant, someone in my family enjoyed playing with me, and I enjoyed it too
- When I was a child, there were relatives in my family who helped me feel better when I was sad or worried
- When I was a child, neighbors or my friends’ parents seemed to like me
- When I was a child, teachers, coaches, youth leaders, or ministers were there to help me
- Someone in my family cared about how I was doing in school
- My family, friends, and neighbors talked about making our lives better
- We had rules in our house and were expected to keep them
- When I felt really bad, I could almost always find someone I trusted to talk to
- As a youth, people notice that I was capable and could get things done
- I was independent and a go-getter
- I believe that life is what you make it
- There are people I can count on in my life
- There are other factors in my life that have or do make me resilient in the face of hardship
  - What are these other factors? __________
- I am having trouble feeling resilient right now

What types of information do you think might assist you in your child’s health journey? (Please check all that apply)

- Information on transportation
- Parking information or assistance
- Online or print information about other children with similar conditions
- Online information on eligibility for disability supports for my child
- Information on assistance to cover the cost of essential medicines, devices, or supplies
- Information about applying for First Nations’ benefits
- Immigration or new Canadian services
- Other __________

Is there anything your doctors or the hospital could do to make this visit easier on you and your family?
