# Supplementary material for: Assessing the Social Determinants of Health and Adverse Childhood Experiences in Patients Attending a Children's Hospital Cleft Palate-Craniofacial Program
Source: Cleft Palate Craniofac J. 2021 Nov 3;59(12):1482–9. doi: 10.1177/10556656211048742 (PMC9585543; doi:10.1177/10556656211048742)
Supplement: sj-docx-2-cpc-10.1177_10556656211048742 - Supplemental material for Assessing the Social Determinants of Health and Adverse Childhood Experiences in Patients Attending a Children's Hospital Cleft Palate-Craniofacial Program [file sj-docx-2-cpc-10.1177_10556656211048742.docx]

**BEARS Questionnaire**

If you have a family doctor, nurse practitioner, counselor, or other general health provider, can you turn to them for support for help with disability forms, nutrition/caloric supplements (e.g. Vitamins/Boost), housing transportation-related forms, etc.?

- Yes
- No
- I don’t have a regular health provider

How important is it that your healthcare provider include you in the decisions about your child’s health?

- Very important
- Somewhat important
- Neutral
- Not important
- Not ever an issue

Is it important for you to have a system to track your child’s appointments and/or other health needs?

- Yes
- No
- Not an issue or we don’t need this

If you have a system, please explain. If you don’t, describe what would help. ________________

Do you submit tax forms each year to be considered for your child or disability eligibility?

- Yes
- No
- Unsure

At the end of the month, do you have difficulty making ends meet?

- Never
- Sometimes (a few times per year)
- Always (every month)

Does the cost of essential medication, devices, disposables, or other medical supplies affect your ability to follow prescribed treatment plans, or provide for your child?

- Yes
- No

If yes, how? ___________________________________________________________________

How many people live in your home? _________

How many children (under 19 years old) live in your household? __________

What is your postal code? First 3 characters only. __________

What is your estimated household income?

- $0-$40,000
- $40,000-$80,000
- $80,000-$100,000
- $100,000-$120,000
- $120,000 and over

Have you ever felt excluded or uncomfortable during your child’s healthcare journey because of extra needs, language, culture, gender, or other reasons?

- Yes
- No
- Unsure

If yes, please explain. ____________________________________________________________

Do you or anyone in your household identify as a minority: visually, ethnically, or culturally?

- Yes
- No

Have you ever received extended benefits, child welfare, or other social services for your child or for their health needs?

- Yes
- No
- Unsure

If yes, check all that apply

- Federal Disability Child Tax Benefits (T2201 Disability Tax Credit Certificate filled out by a doctor or other provider)
- Extra-Income supplements (e.g. Income assistance, Employment Insurance (EI))
- Foster Care or Family Preservation Worker
- Child and Youth with Special Needs (CYSN)
- Child and Youth Mental Health (CYMH)
- First Nations, Metis, Inuit, or other Indigenous Health Benefits
- Other __________

Which of the following apply to you? (parents/guardian)

- When I was a child, family members, teacher, coaches, youth leaders, or others were there to help me
- There are people that I can count on now in my life
- I am having a hard time feeling resilient right now

Do you feel there is a supportive network available to help you or your family members in times of need, if needed, at this hospital or nearby? (e.g. spiritual care, quiet spaces, healing circle, social work support, patient navigators, interpreters, etc.)

- Often
- Sometimes
- Rarely

What is your relationship with your child?

- Mother
- Father
- Grandparents
- Other Family Member (sister, aunt, uncle, etc.)
- Foster parent
- Social Worker
- Other __________

In times of stress, how many people can you turn to for support? (e.g. friends, partner, parents, grown children, neighbours, elders, spiritual/religious guide, teacher, coach, health nurse, doctor, co-worker, etc.)

- Fewer than 4
- 4-8
- 9-13
- 14-19
- More than 20

*Optional – ACEs*

*Childhood experiences, both positive and negative, can impact lifelong health and future opportunities. Much of the expert research in this area is referred to as Adverse Childhood Experiences (ACEs). Many children experience stressful life events that can affect their health and wellbeing. The results from this questionnaire will assist your child’s health care team in assessing health and determining guidance.*

*This part of the questionnaire asks some personal questions about longer-term health risks associated with exposure to stress and ACEs. Our healthcare team believes that recognizing risks is an opportunity for us to address and help you prevent future health problems.*

*Please read the following statements. Please DO NOT mark or indicate which specific statements apply to your child. We recognize that the following questions are sensitive. This section is optional. You can also choose not to continue.*

Count the number of statements that apply to your child and write the total number in the box provided. __________

At any point since your child was born…

- Your child experienced harassment or bullying at school
- You child lived with a parent or grandparent who died
- Your child was separated from their primary care giver during deportation or immigration
- Your child was in foster care
- Your child had a serious medical procedure or life-threatening illness
- Your child often saw or heard violence in their neighbourhood
- Your child was often treated badly because of race, sexual orientation, place of birth, disability, or beliefs

Count the number of statements that apply to your child and write the total number in the box provided. __________

At any point since your child was born…

- Your child’s parents or guardians were separated or divorced
- Your child lived with a household member who served time in jail or prison
- Your child lived with a household member who was depressed, mentally ill, or attempted suicide
- Your child saw or heard household members hurt or threated to hurt each other
- A household member swore at, insulted, humiliated, or put down your child in a way that scared your child OR a household member acted in a way that made your child afraid that s/he might be physically hurt
- Someone touched your child’s private parts or asked to touch their private parts in a sexual way
- More than once, your child went without food, clothing, a place to live, or had no one to protect her/him
- Someone pushed, grabbed, slapped, or threw something at your child OR your child was hit so hard that your child was injured or had marks
- Your child lived with someone who had a problem with drinking or using drugs
- Your child often felt unsupported, unloved, and/or unprotected

Extra space for responding to earlier questions. _______________________________________

Is there anything your doctors or this clinic could do to support you or make this visit easier on you and your family? Do you have any suggestions to improve this survey? You may also write directly on the questions. _________________________________________________________
